# Foot cues can elicit covert orienting of attention

**DOI:** 10.1007/s00426-023-01827-7

**Published:** 2023-04-14

**Authors:** Mario Dalmaso

**Affiliations:** Department of Developmental and Social Psychology, University of Padova, Via Venezia 8, 35131 Padua, Italy

## Abstract

Humans tend to orient their attentional resources towards the same location indicated by spatial signals coming from the others, such as pointing fingers, head turns, or eye-gaze. Here, two experiments investigated whether an attentional orienting response can be elicited even by foot cues. Participants were asked to localize a peripheral target while a task-irrelevant picture of a naked human foot, oriented leftward or rightward, was presented on the centre of the screen. The foot appeared in a neutral posture (i.e., standing upright) or an action-oriented posture (i.e., walking/running). In Experiment 1, neutral and action-oriented feet were presented in two distinct blocks, while in Experiment 2 they were presented intermixed. The results showed that the action-oriented foot, but not the neutral one, elicited an orienting response, though this only emerged in Experiment 2. This work suggests that attentional shifts can be induced by action-oriented foot cues, as long as these stimuli are made contextually salient.

## Introduction

A broad and increasing body of literature has shown that people tend to orient their attention towards the same location indicated by a variety of spatial signals provided by their conspecifics, such as eye-gaze direction (Dalmaso et al., 2020c; Frischen et al., 2007; McKay et al., 2021), pointing gestures (Ariga & Watanabe, 2009; Langton & Bruce, 2000), and head and body turns (Azarian et al., 2017; Langton & Bruce, 1999). The ability to pay attention to the same location as another individual, which is often referred to as ‘social attention’ (Kingstone, 2009), is essential in daily-life social interactions, as it allows people to establish fluent relationships and interactions with both others and the physical environment in which they act (Capozzi & Ristic, 2018; Emery, 2000).

The impact of social stimuli on visual orienting of attention has generally been investigated by adopting spatial cueing tasks derived from the paradigm proposed by Posner (1980). In these tasks, participants are initially presented with a task-irrelevant social cue, communicating a spatial direction (e.g., a pointing finger oriented rightwards), and appearing at a central position. After a variable stimulus onset asynchrony (SOA), they are asked to provide a motor response (e.g., a key press) to a target appearing in the periphery. Behavioural advantages (i.e., shorter reaction time and greater accuracy) are generally observed when the target appears at a spatial location indicated by the cue (i.e., a congruent trial) than when it appears at a different spatial location (i.e., an incongruent trial; see, e.g., McKay et al., 2021).

It is interesting to note that some studies have also reported attentional orienting in response to stimuli related to the feet, suggesting that our social attention system would be sensitive even to spatial signals coming from the lower part of the body of others. Most studies focused on the biological motion associated with feet and presented participants with highly impoverished stimuli consisting of moving light dots that mimic the movement of an individual (Bardi et al., 2015; Troje & Westhoff, 2006; Wang et al., 2014). For instance, in Wang et al. (2014), participants were engaged in a spatial cueing task in which two moving light dots, obtained by tracking the walking of a real individual with motion markers attached to his ankles, were used as cues, and the task consisted of localising (with a manual response) a peripheral target appearing 600 ms after cue onset. Evidence of a reliable orienting of attention emerged.

Other studies used more concrete and ecological foot-related stimuli, reporting mixed results. On the one hand, a recent study (Dalmaso, 2023) presented participants with footprint stimuli, which represent an indirect sign of the passage of another person within the environment. In this case, different types of footprint stimuli (i.e., human barefoot, shoes, and animal footprints) were used as central spatial cues, while participants were asked to discriminate (with a manual response) a peripheral target that appeared after a variable SOA (i.e., 200, 600, or 1000 ms). Evidence of a reliable orienting of attention emerged regardless of the type of cue, suggesting that humans are sensitive to footprints of different natures.

On the other hand, and of particular interest for the present work, another recent study (Chen et al., 2020) employed a spatial cueing task in which the picture of a human hand with the index finger pointing left or right, and the picture of a naked human foot oriented leftward or rightward, were used as spatial cues and presented in two distinct blocks of trials. After either 100 or 1000 ms, a peripheral target appeared that was to be localised with either a manual or foot response. A reliable orienting of attention emerged for the pointing finger but not for the foot stimulus, and this emerged regardless of the responding effector. Chen et al. (2020) concluded that the lack of an attentional orienting effect for the foot stimulus could reflect the fact that, in our everyday social interactions, we scarcely use our feet to convey a message of spatial nature, which is something that certainly cannot be said for finger-pointing stimuli. Furthermore, Chen et al. (2020) also suggested that the foot stimulus used in their study belonged to a stationary person (i.e., an individual standing upright) and was therefore not associated with a clear and strong pointing position. Indeed, the direction of the feet of person standing upright does not necessarily indicate that such an individual is paying attention in the same direction pointed her/his feet are pointed, or that, more generally, she/he is interested in such a spatial direction (i.e., during a face-to-face interaction, our feet can be directed towards a variety of different spatial locations without necessarily having a specific spatial meaning). On the other hand, the foot stimuli associated with an action-oriented posture (e.g., walking/running) would be a more direct and less ambiguous index of spatial pointing. Therefore, a reliable orienting response to foot stimuli could be expected for action-oriented foot stimuli. This possibility was tested here.

## The present study

The current study, inspired by the work of Chen et al. (2020), represents a further attempt to reveal covert orienting of attention in response to the picture of a real foot. Participants were presented with the picture of a naked human foot oriented leftward or rightward. The foot cue belonged to a person in a neutral posture (i.e., standing upright), as in Chen et al. (2020), or in an action-oriented posture (i.e., walking/running). The hypothesis was straightforward: If it is true that a clear and strong pointing position associated with feet is a key element to elicit an attention-orienting response, a reliable orienting of attention was therefore expected for the foot stimulus associated with an action-oriented posture, but not for the foot stimulus associated with a neutral posture (in line with Chen et al., 2020).

## Experiment 1

The purpose of Experiment 1 was to extend the results reported by Chen et al. (2020). For this reason, the task was similar to that employed by Chen et al. (2020): Participants were presented with task-irrelevant central foot stimuli associated with a neutral or an action-oriented posture, while a target to be localised appeared in the periphery. To adhere as much as possible to the design adopted by Chen et al. (2020), the two types of stimuli (neutral versus action-oriented foot cues) were presented in two distinct blocks.

### Method

#### Participants

The sample size was calculated a priori with the Superpower R package (Lakens & Caldwell, 2021). A simulation-based power analysis (power = 80%, α = 0.05, 10,000 simulations) was performed for a 2 (congruency) × 3 (SOA) × 2 (cue) ANOVA design (see “Results”), with the aim of detecting a reliable congruency × cue interaction, as well as a reliable congruency effect for the action-oriented foot cue. The simulation was based on the data (i.e., means, standard deviations, and the correlations) provided by Dalmaso (2023; Experiment 1), which represents the closest comparison with the experimental approach adopted in the current study (i.e., an online behavioural task with foot-related stimuli; see “Apparatus, stimuli, and procedure”). This analysis indicated that a sample size of 38 was necessary. A greater number of participants were contacted to deal with potential withdrawals. The final sample was made up of 42 naïve participants *(M* = 22 years, SD = 2.05; 14 males, 4 left-handed; handedness was self-reported by participants), who took part on a voluntary basis. They were recruited (via email) within the student population of the University of Padova. Informed consent was obtained before the experiment and the study, approved by the Ethics Committee for Psychological Research of the University of Padova, was conducted in accordance with the Declaration of Helsinki.

#### Apparatus, stimuli, and procedure

**Fig. 1:**
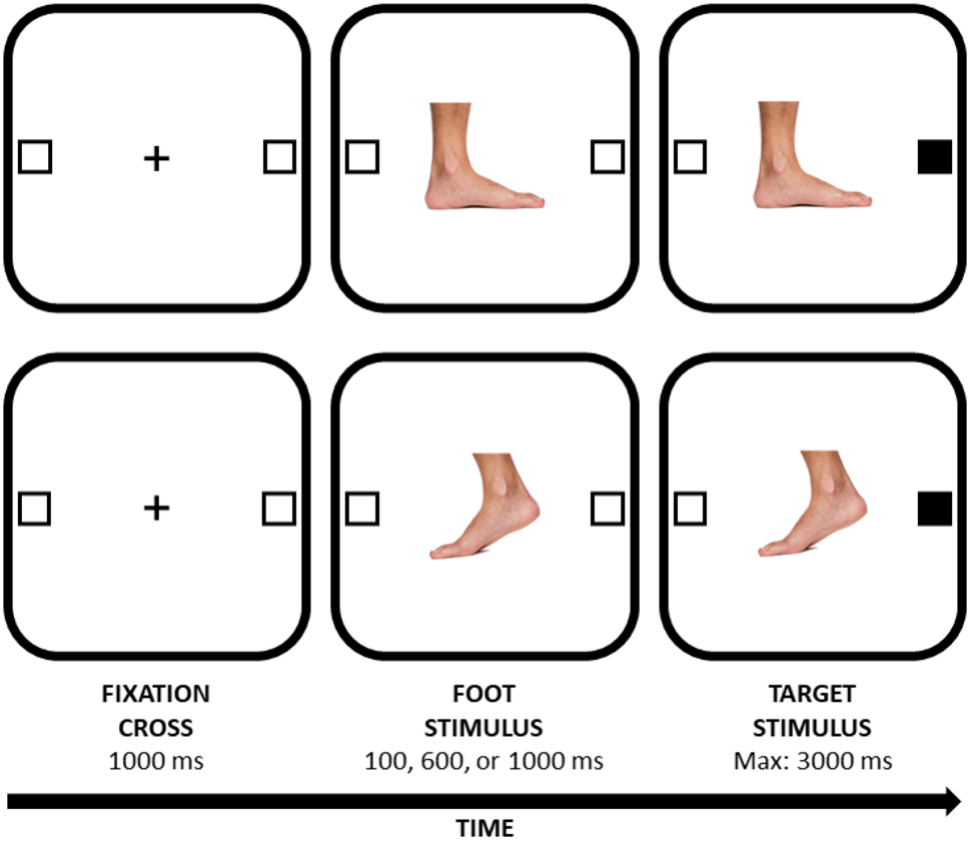
Examples of trials used in both Experiments 1 and 2. Stimuli are not drawn to scale. The upper panel illustrates a congruent trial in which a foot associated with a neutral posture is oriented towards the same spatial location (i.e., right) of the upcoming target. The lower panel illustrates an incongruent trial in which a foot associated with an action-oriented posture is pointed toward the opposite spatial location (i.e., left) of the upcoming target

PsychoPy was used to develop the experiment, which was then delivered online using the Pavlovia platform (Peirce et al., 2019). These software programs allow the collection of reliable and precise data across a variety of operating systems and web browsers (Bridges et al., 2020).^1^ Both the stimuli and the procedure were similar to those used by Chen et al. (2020) and are described in Fig. 1. Neutral and action-oriented foot cues were presented in two distinct blocks with the block order counterbalanced across participants. At the beginning of each trial, a black fixation cross (Arial font, 0.1 normalised units) appeared at the centre of the screen for 1000 ms. At the same time, two placeholders appeared, namely two white boxes with a black border (width 40 px; height 40 px), both on the left and on the right (i.e., ± 0.8 normalised units) with respect to the centre of the screen, and remained visible for the duration of the trial. Then, the central picture of a foot (width 300 px; height 253 px), oriented either leftward or rightward with same probability (i.e., 50%), appeared at the centre of the screen. After an SOA of 100, 600, or 1000 ms,^2^ the inner colour of one of the two placeholders changed from white to black, acting as the target stimulus. Participants were instructed to detect, as fast and accurately as possible, the position of the target by pressing a response key (the D key to indicate left, the K key to indicate right). They were also told to ignore the foot image, since it did not predict the spatial location of the upcoming target, and to look at the centre of the screen for the whole duration of the trial. A trial ended when a response was provided or 3000 ms elapsed, whichever came first. Additionally, missed and wrong responses were signalled with feedback (i.e., the black words ‘MISSED RESPONSE’ or ‘ERROR,’ respectively in Arial font, 0.1 normalised units) that appeared centrally, for 500 ms. All stimuli were presented on a white background. A practice block of 10 trials was followed by the two experimental blocks of 144 trials each. Therefore, each participant responded to 288 experimental trials in total. Within the two experimental blocks, each experimental condition appeared for an equal number of times (i.e., 24 trials per condition) and in a random order.

### Results

Trials with missed (0.09% of trials) or incorrect (0.50% of trials) responses were rare, and were discarded without further analysis. Trials with a correct response and with a latency smaller than 100 ms or greater than 1000 ms (0.71% of trials) were also discarded, following the same procedure used by Chen et al. (2020).

Mean reaction times (RT) of correct trials were analysed through a repeated-measures ANOVA with congruency (2: congruent vs. incongruent), SOA (3: 100 vs. 600 vs. 1000 ms), and cue (2: neutral vs. action-oriented) as within-participant factors. The Bonferroni correction was used to adjust for multiple comparisons.

**Fig. 2:**
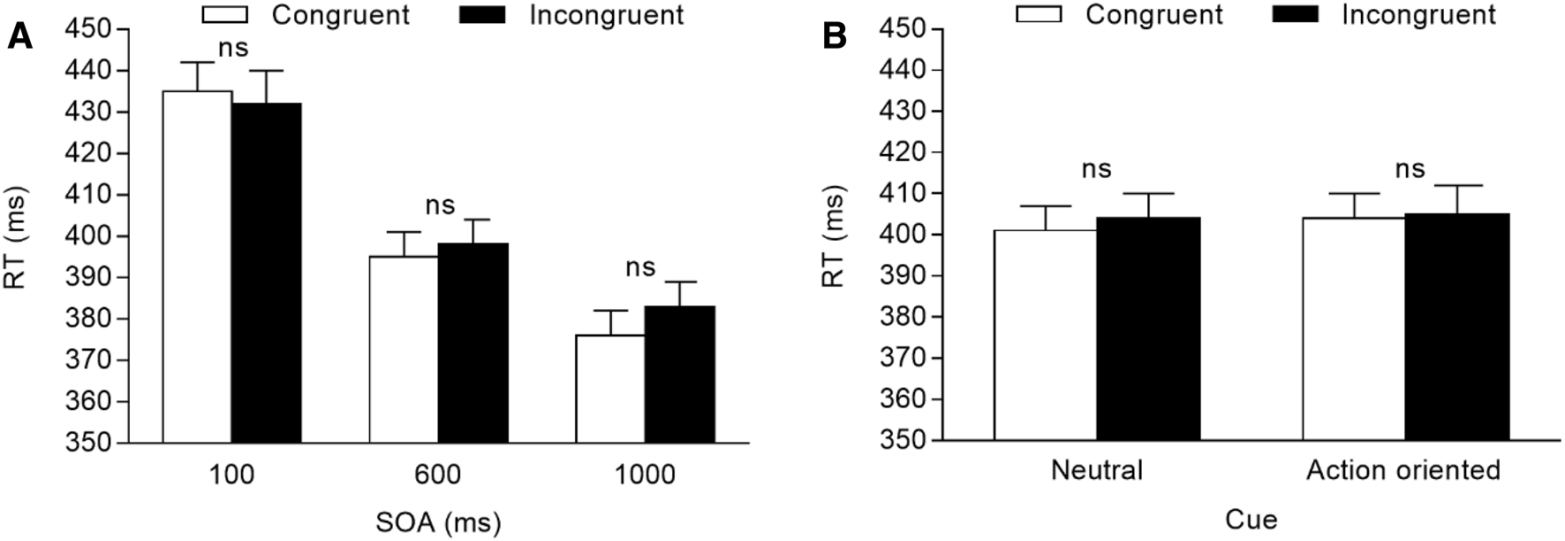
Mean RTs for congruent vs. incongruent trials observed in Experiment 1. **A** Mean RTs (ms) for congruent vs. incongruent trials, separated by SOA. **B** Mean RTs (ms) for congruent vs. incongruent trials, separated by cue. Error bars are SEM. *ns* non-significant comparison

The main effect of congruency was not significant, *F*(1, 41) = 1.936, *p* = 0.172, *η*^2^_*p*_ = 0.045, while the main effect of SOA was significant, *F*(2, 82) = 183.419, *p* < 0.001, *η*^2^_*p*_ = 0.817, indicating that RTs were slowest at the 100 ms SOA (434 ms), intermediate at the 600 ms SOA (397 ms), and fastest at the 1000 ms SOA (380 ms). The congruency × SOA interaction was also significant, *F*(2, 82) = 3.825, *p* = 0.026, *η*^2^_*p*_ = 0.085. The interaction was further analysed with two-tailed paired t tests comparing congruent and incongruent trials separately for each SOA, showing that a congruency effect did not emerge at the two shorter SOAs (*p*s > 0.717), as well as at the longest SOA (*p* = 0.075), although the means showed an increasing trend in the magnitude of the attention-orienting response (see also Fig. 2A). Importantly, the predicted congruency × cue interaction was not significant, *F*(1, 41) = 0.580, *p* = 0.451, *η*^2^_*p*_ = 0.014. For completeness, two-tailed paired t tests comparing congruent and incongruent trials separately for each cue were not significant (*p*s > 0.220; see Fig. 2B). No other results were significant (*p* > 0.463), including the congruency × SOA × cue interaction (*p* = 0.207; see Table 1).^3^

**Table 1:** Mean RTs and SEM for all experimental conditions in Experiments 1 and 2

	Neutral						Action oriented					
	100 ms		600 ms		1000 ms		100 ms		600 ms		1000 ms	
	C	I	C	I	C	I	C	I	C	I	C	I
Exp. 1												
Mean	437	432	392	398	374	382	433	433	399	399	379	384
SEM	7.09	8.07	5.92	6.36	5.98	6.23	7.69	8.35	6.07	7.00	6.72	7.4
Exp. 2												
Mean	437	429	392	392	371	375	433	431	386	395	367	376
SEM	9.34	9.72	8.10	8.02	7.36	8.58	9.15	9.51	7.72	8.64	6.98	7.87

Mean RTs (in ms) and SEM for all the experimental conditions observed in Experiment 1 and 2

*C* congruent, *I* incongruent

### Discussion

Overall, the main results emerging from Experiment 1 recall those observed by Chen et al. (2020). In fact, there was no evidence of a reliable attention-orienting response, confirmed by the absence of the main effect of congruency, and this absence was also reflected at the different levels of SOA. Of particular interest, the predicted congruency × cue interaction was also not significant, indicating that the action-oriented foot stimuli—like the neutral foot stimuli—had no impact on the social attention system. This unexpected result was further explored in Experiment 2, in which the saliency of the action-oriented foot cue was increased in an attempt to reveal a magnified orienting response for such a stimulus.

## Experiment 2

The rationale underlying Experiment 2 was based on research on social attention showing that a modulatory role in visual orienting of a social variable can be observed when different types of social stimuli are presented in an intermixed rather than blocked fashion (see, e.g., Dalmaso et al., 2020a; Pavan et al., 2011; Zhang et al., 2023). The general idea underlying this approach is that a given stimulus can be less or more relevant to participants if it is presented with a concurrent comparative stimulus (Carvalho & Goldstone, 2017). As for the social attention literature, for example, Zhang et al. (2023) reported a greater gaze-cueing effect—the covert orienting of attention elicited by averted-gaze faces—for faces belonging to White individuals (vs. Asian individuals), but only when these two types of faces were presented intermixed. Indeed, when the two types of faces were presented in two distinct blocks of trials, the magnitude of gaze-mediated orienting was not modulated by face type. The intermixed presentation would favour the activation of categorization processes based on a continuous comparison of different types of stimuli, while this comparison is, by definition, lacking when a blocked presentation is adopted. A similar rationale could also be applied to the present context. More precisely, the block design adopted in Experiment 1 suggested that our social attention system treated the two types of foot cues similarly. However, and in line with previous studies (Dalmaso et al., 2020a; Pavan et al., 2011; Zhang et al., 2023), an intermixed presentation of stimuli could increase the saliency of the action-oriented foot cue over the neutral foot cue, thus favouring the emergence of an orienting response for the former cue. Therefore, this possibility was examined in Experiment 2.

### Method

#### Participants

The sample size was intended to be identical to that used for Experiment 1. Therefore, a new sample of 42 naïve individuals (*M* = 23 years, SD = 3, 15 males, 5 left-handed; handedness was self-reported by participants) was tested. They took part on a voluntary basis and were recruited within the student population of the University of Padova. Informed consent was obtained before the experiment and the study, approved by the Ethics Committee for Psychological Research of the University of Padova, was conducted in accordance with the Declaration of Helsinki.

#### Apparatus, stimuli, and procedure

Everything was identical to Experiment 1, with the only exception that the two types of foot stimuli (i.e., neutral vs. action-oriented) were presented intermixed rather than into two distinct blocks of trials.

### Results

Data were analysed as in Experiment 1. Trials with missed (0.06% of trials) or incorrect (0.62% of trials) responses were rare, and were discarded without further analysis. Trials with a correct response and with a latency smaller than 100 ms or greater than 1000 ms (0.37% of trials) were also discarded.

**Fig. 3:**
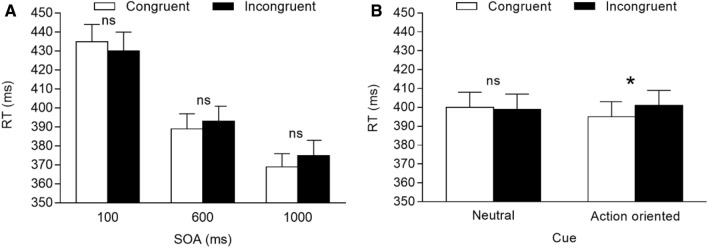
Mean RTs for congruent vs. incongruent trials observed in Experiment 2. **A** Mean RTs (ms) for congruent vs. incongruent trials, separated by SOA. **B** Mean RTs (ms) for congruent vs. incongruent trials, separated by cue. Error bars are SEM. *ns* non-significant comparison; *Significant comparison

The main effect of congruency was not significant, *F*(1, 41) = 1.739, *p* = 0.195, *η*^2^_*p*_ = 0.041, while the main effect of SOA was significant, *F*(2, 82) = 183.122, *p* < 0.001, *η*^2^_*p*_ = 0.817, indicating that RTs were slowest at the 100 ms SOA (432 ms), intermediate at the 600 ms SOA (391 ms), and fastest at the 1000 ms SOA (372 ms). The congruency × SOA interaction was also significant, *F*(2, 82) = 5.513, *p* = 0.006, *η*^2^_*p*_ = 0.119. The interaction was further analysed with two-tailed paired t tests comparing congruent and incongruent trials separately for each SOA, showing that a congruency effect did not emerge at the two shorter SOAs (*p*s > 0.183), as well as at the longest SOA (*p* = 0.060), although the means showed an increasing trend in the magnitude of the attention-orienting response (see also Fig. 3A). More importantly, the predicted congruency × cue interaction was also significant, *F*(1, 41) = 5.948, *p* = 0.019, *η*^2^_*p*_ = 0.127. The interaction was further analysed through two-tailed paired t tests comparing congruent and incongruent trials for each cue, showing that the attentional orienting was not significant for the neutral foot, *t*(41) = 0.729, *p* = 0.940, *d* = 0.112, but was significant for the action-oriented foot, *t*(41) = 2.833, *p* = 0.014, *d* = 0.437 (see Fig. 3B). No other results were significant (*p*s > 0.267), including the congruency × SOA × cue interaction (*p* = 0.807; see Table 1).^4^

Finally, as suggested by a reviewer, explorative analyses were also performed on a sub-sample of trials relative to the action-oriented foot cues. These trials were classified as follows: (a) trials in which an action-oriented cue was preceded by a neutral cue pointing towards the same direction and (b) trials in which an action-oriented cue was preceded by a neutral cue pointing towards the opposite direction. These analyses were designed to test the possible role of apparent motion on the orienting response. The only significant results were the main effect of SOA (*p* < 0.001) and the congruency × type of trial (2: ‘a’ vs. ‘b’) interaction (*p* = 0.035). Bonferroni-corrected comparisons showed that attentional orienting emerged in ‘b’ (*p* = 0.01) but not in ‘a’ (*p* = 0.956).

### Discussion

The results of Experiment 2 partially confirmed those reported in Experiment 1. Indeed, also in this case, the main effect of congruency was not significant, and the absence of an attention-orienting response was also reflected at the different levels of SOA. However, differently from Experiment 1, the expected congruency × cue interaction was significant, indicating that a reliable orienting of attention emerged for the action-oriented foot cue but not for the neutral foot cue. This last finding provides supporting evidence for the idea that the intermixed presentation of stimuli may have increased the saliency of the action-related foot cues over the neutral ones, thus favouring an attention-orienting response in the former case.

## General discussion

This work explored whether the picture of a human foot, oriented leftwards or rightwards, can elicit attentional orienting towards the same spatial location in an observer. In two experiments, participants were required to locate a peripheral target while neutral posture and action-oriented foot cues appeared in the centre of the screen in a blocked (Experiment 1) or intermixed (Experiment 2) fashion. The main result showed that the predicted congruency × cue interaction was significant only in Experiment 2, with subsequent comparisons indicating that reliable attention-orienting emerged for the action-oriented foot cue but not for the neutral foot cue. Overall, the results emerging from the present study provide an extension to what was reported by Chen et al. (2020) and, more generally, provide additional supporting evidence for the notion that foot-related stimuli can impact the social attention system (Dalmaso, 2023; Wang et al., 2014).

A consistent result between the two experiments was the presence of a significant congruency × SOA interaction, although none of the subsequent comparisons revealed a statistically significant attention-orienting response for foot stimuli. However, it should be noted with caution that the means seem to suggest an increase in the magnitude of the attention-orienting response effect with SOA. Even if the link between social orienting and SOA is still a matter of debate in the social attention literature, the available evidence indicates that social orienting would diminish at relatively long SOAs. This is what was observed in recent meta-analytic work on the gaze-cueing effect (McKay et al., 2021), according to which the magnitude of such an effect would be higher for relatively short SOAs (e.g., smaller than 200 ms until about 600–800 ms), then diminished at relatively longer SOAs (i.e., after about 800 ms). Nevertheless, it is important to keep in mind that eye-gaze stimuli are likely the most relevant spatial signals for humans, and evolution seems to have developed specific neurocognitive systems dedicated to eye-gaze processing and gaze-mediated orienting (Emery, 2000). Therefore, the high relevance of eye-gaze stimuli could explain the possible behavioural differences that could emerge between eye-gaze and other social signals that convey spatial direction. Additional studies are needed to address the temporal dynamics underlying the attention-orienting response to different types of social stimuli.

The results of Chen et al. (2020) and those reported here suggest that the orienting response elicited by foot stimuli is less robust and automatic compared to the orienting response elicited by cues that are more commonly used to guide others’ attention, such as eye-gaze or pointing gestures. In this regard, a recent theoretical framework called *eyeTUNE* (Dalmaso et al., 2020c) has been proposed to describe the possible social modulations of the gaze-cueing effect. According to this framework, an orienting response to eye-gaze stimuli would represent a default condition due to the great relevance of such stimuli for human beings. In fact, following the direction of another’s gaze could help identify a possible relevant stimulus in the environment, and therefore gaze-mediated orienting should appear by default. This would explain why reliable gaze-cueing effects have been documented for a variety of different eye-gaze stimuli, including simple (and socially impoverished) cues such as schematic eyes (Friesen & Kingstone, 1998) and at different levels of SOAs, including extremely very brief durations such as 14 ms (Hietanen & Leppänen, 2003). In sum, the picture emerging from Chen et al. (2020) and the present study suggests that at least two factors play a crucial role in the emergence of an attentional response to foot cues, which are (1) the presence of a cue with an evident action-oriented nature and (2) the adoption of a context (i.e., an intermixed presentation of stimuli) designed to incentivize a continuous comparison between neutral and action-oriented cues and favouring an increment in the saliency of the latter. Furthermore, the results provided by the analyses carried out on a sub-sample of trials of Experiment 2 revealed the presence of a peculiar trial-by-trial modulation, according to which the spatial nature of the *n*-1 trial influenced the performance on the *n* trial (see also, e.g., Ciardo et al., 2019). This modulation could be related to apparent motion, which likely appeared to participants as particularly evident when an action-oriented cue was preceded by a neutral cue pointing towards the opposite direction. However, given the explorative nature of these analyses, and the fact that the role of trial order is still a largely unexplored topic in social attention, future studies are needed to get a precise overview of such modulations in the present context.

One may wonder whether the experimental setting employed in Experiments 1 and 2 is suitable for detecting gaze-cueing of attention. Therefore, a follow-up experiment was conducted, which was similar to Experiments 1 and 2 but with averted-gaze faces instead of foot cues.^5^ A reliable gaze-cueing effect was found, thus confirming previous studies (e.g., Friesen & Kingstone, 1998; for other studies investigating gaze-mediated orienting through online tasks, see also, e.g., Dalmaso et al., 2021a, 2021b; Gregory, 2022; Villani et al., 2022). Furthermore, exploratory analyses compared the magnitude of the gaze-cueing effect with the magnitude of the orienting response elicited by the action-oriented foot cues in Experiment 3, but no differences emerged. This aligns with previous evidence showing that, at least at the behavioural level, gaze cues can elicit a similar orienting response as compared to other social cues, such as, for instance, pointing gestures (e.g., Cazzato et al., 2012; Dalmaso et al., 2015).

Future studies should further test the boundary conditions linking social attention and foot stimuli, for example, by employing different tasks and measures (e.g., eye movements; Dalmaso et al., 2020b; Kuhn & Kingstone, 2009) that are known to tap into attentional mechanisms from alternative perspectives (see also Hommel et al., 2019). In addition, even if in Chen et al. (2020) participants were immersed in an experimental context similar to that employed here, it is important to recall that in that work responses were provided with the hands or the feet on different blocks of trials. Since there is evidence showing that pairing different types of responses could activate different attentional mechanisms with pairs of responses within a trial (see, e.g., Taylor & Klein, 2000; Hilchey et al., 2012), another avenue for future work could be to manipulate response context (e.g., hand vs. foot responses) and the contexts concerning stimulus type and presentation (e.g., intermixed vs. blocked presentation of neutral vs. action-related foot cues).

The present work represents an additional piece of evidence showing that the modulatory role of a given variable becomes evident when an intermixed, rather than blocked, presentation of different stimuli is adopted, which is in line with some previous studies on the gaze-cueing effect (e.g., Dalmaso et al., 2020a; Pavan et al., 2011; Zhang et al., 2023). Additional, albeit indirect, evidence for this notion can also be found in studies examining spatial orientation in response to body pictures depicting individuals with neutral or action-oriented postures (Azarian et al., 2016, 2017). Indeed, in Azarian et al. (2016), evidence for an orienting response emerged for bodies (oriented leftwards or rightwards) with a posture communicating a threat (fear or anger), but not for bodies with a neutral posture. However, in a subsequent follow-up study, Azarian et al. (2017) found that bodies with a neutral posture can also elicit a reliable orienting response. The authors posited that this might be due to the fact that while in Azarian et al. (2016) all stimuli were presented intermixed (thus leading to the emergence of a comparative context which would have favored the processing of the threat stimuli over the neutral ones), in Azarian et al. (2017) only neutral stimuli were employed, thus avoiding any comparison with other types of stimuli. In sum, the picture emerging from social attention literature clearly indicates that the mechanisms governing the attentional response to different social stimuli appears to be influenced by the interplay between the specific nature of the stimuli and the context in which such stimuli are presented.

### Conclusion

This work extends our knowledge about social attention, showing that even foot cues are capable of eliciting an attention-orienting response in an observer, provided that certain requirements are met.

## Data Availability

Stimuli, raw and processed data are available in the Open Science Framework repository, https://doi.org/10.17605/OSF.IO/XCMFV.

## Footnotes

1 Recent studies have also shown that when a task is delivered online or conducted in a laboratory, it leads to a similar pattern of results (see, e.g., Vicovaro et al., 2022).

2 Three different SOAs were used to assess the temporal development of the cueing effect for foot stimuli, if any. Indeed, if relatively-short SOAs—such as less than 300 ms—can be associated with a more reflexive orienting, relatively longer SOAs can permit more volitional mechanisms to emerge (Müller & Rabbitt, 1989).

3 Exploratory analyses were also performed to test whether performance differed between the first and second block of trials. The factor Block did not lead to any significant result (*p*s > .125).

4 Exploratory analyses were also performed to test whether performance differed between the first and second block of trials. Only the theoretically irrelevant interaction between SOA and Block emerged (*p* = .049; all other *p*s > .334). In addition, exploratory analyses were also performed to compare the two experiments. Hence, the data from Experiments 1 and 2 were combined, and an additional factor called experiment (2: 1 vs. 2) was added as a between-participant factor. Importantly, the congruency × cue × experiment interaction was significant, *F*(1, 82) = 5.525, *p* = .021, *η*^2^_*p*_ = .063, indicating that the performance was actually different between the two experiments. No other significant results involving the factor experiment emerged from the analyses (*p*s > .311).

5 A new sample of 42 naïve individuals (*M* = 25 years, SD = 2.30, 27 males, 8 left-handed; handedness was self-reported by participants), recruited on a voluntary basis within the student population of the University of Padova, was tested. Data were analysed as in Experiments 1 and 2. Trials with missed (0.04% of trials) or incorrect (0.77% of trials) responses were rare, and were discarded without further analysis. Trials with a correct response and with a latency smaller than 100 ms or greater than 1000 ms (0.59% of trials) were also discarded. The main effect of congruency was significant, *F*(1, 41) = 10.786, *p* = .002, *η*^2^_*p*_ = .208, indicating that RTs were smaller on congruent trials (*M* = 385 ms, SE = 6.5) than on incongruent trials (*M* = 391 ms, SE = 6.67). The main effect of SOA was also significant, *F*(2, 82) = 254.823, *p* < .001, *η*^2^_*p*_ = .861, indicating that RTs were slowest at the 100 ms SOA (419 ms), intermediate at the 600 ms SOA (386 ms), and fastest at the 1000 ms SOA (358 ms). The congruency × SOA interaction was not significant, *F*(2, 82) = .978, *p* = .380, *η*^2^_*p*_ = .023. In addition, the magnitude of the gaze-cueing effect was compared with the magnitude of the cueing effect that emerged from the action-oriented foot cue in Experiment 3. The magnitude of the cueing effects was calculated as in previous studies (i.e., RTs on incongruent trials—RTs on congruent trials; see, e.g., Driver et al., 1999). Data were analysed with an independent sample t test, showing that no differences emerged (*p* = .878).

## References

[CR1] Ariga A, Watanabe K (2009). What is special about the index finger?: The index finger advantage in manipulating reflexive attentional shift. Japanese Psychological Research.

[CR2] Azarian B, Buzzell GA, Esser EG, Dornstauder A, Peterson MS (2017). Averted body postures facilitate orienting of the eyes. Acta Psychologica.

[CR3] Azarian B, Esser EG, Peterson MS (2016). Watch out! Directional threat-related postures cue attention and the eyes. Cognition and Emotion.

[CR4] Bardi L, Di Giorgio E, Troje NF, Simion F (2015). Walking direction triggers visuo-spatial orienting in 6-month-old infants and adults: An eye tracking study. Cognition.

[CR5] Bridges D, Pitiot A, MacAskill MR, Peirce JW (2020). The timing mega-study: comparing a range of experiment generators, both lab-based and online. PeerJ.

[CR6] Capozzi F, Ristic J (2018). How attention gates social interactions. Annals of the New York Academy of Sciences.

[CR7] Carvalho PF, Goldstone RL (2017). The sequence of study changes what information is attended to, encoded, and remembered during category learning. Journal of Experimental Psychology: Learning Memory and Cognition.

[CR8] Cazzato V, Macaluso E, Crostella F, Aglioti SM (2012). Mapping reflexive shifts of attention in eye-centered and hand-centered coordinate systems. Human Brain Mapping.

[CR9] Chen MMZ, Karlinsky A, Welsh TN (2020). Hand, but not foot, cues generate increases in salience at the pointed-at location. Acta Psychologica.

[CR10] Ciardo F, Ricciardelli P, Iani C (2019). Trial-by-trial modulations in the orienting of attention elicited by gaze and arrow cues. Quarterly Journal of Experimental Psychology.

[CR11] Dalmaso, M. (2023). Following others’ traces: Unpredictive footprint cues elicit covert orienting of attention. Manuscript submitted for publication 10.1007/s00426-023-01827-7 PMC10497641 37059960

[CR12] Dalmaso M, Alessi G, Castelli L, Galfano G (2020). Eye contact boosts the reflexive component of overt gaze following. Scientific Reports.

[CR13] Dalmaso M, Castelli L, Franchetti L, Carli L, Todisco P, Palomba D, Galfano G (2015). Altered orienting of attention in anorexia nervosa. Psychiatry Research.

[CR14] Dalmaso M, Castelli L, Galfano G (2020). Early saccade planning cannot override oculomotor interference elicited by gaze and arrow distractors. Psychonomic Bulletin & Review.

[CR15] Dalmaso M, Castelli L, Galfano G (2020). Social modulators of gaze-mediated orienting of attention: A review. Psychonomic Bulletin & Review.

[CR16] Dalmaso M, Castelli L, Galfano G (2021). Increased gaze cueing of attention during COVID-19 lockdown. iScience.

[CR17] Dalmaso M, Zhang X, Galfano G, Castelli L (2021). Face masks do not alter gaze cueing of attention: Evidence from the COVID-19 pandemic. i-Perception.

[CR01] Driver, J., Davis, G., Ricciardelli, P., Kidd, P., Maxwell, E., & Baron-Cohen, S. (1999). Gaze perception triggers reflexive visuospatial orienting. *Visual Cognition, 6*(5), 509–540. 10.1080/135062899394920

[CR18] Emery NJ (2000). The eyes have it: The neuroethology, function and evolution of social gaze. Neuroscience & Biobehavioral Reviews.

[CR19] Friesen CK, Kingstone A (1998). The eyes have it! Reflexive orienting is triggered by nonpredictive gaze. Psychonomic Bulletin & Review.

[CR20] Frischen A, Bayliss AP, Tipper SP (2007). Gaze cueing of attention: Visual attention, social cognition, and individual differences. Psychological Bulletin.

[CR21] Gregory SEA (2022). Investigating facilitatory versus inhibitory effects of dynamic social and non-social cues on attention in a realistic space. Psychological Research Psychologische Forschung.

[CR22] Hietanen JK, Leppänen JM (2003). Does facial expression affect attention orienting by gaze direction cues?. Journal of Experimental Psychology: Human Perception and Performance.

[CR23] Hilchey MD, Klein RM, Ivanoff J (2012). Perceptual and motor inhibition of return: Components or flavors?. Attention, Perception, & Psychophysics.

[CR24] Hommel B, Chapman CS, Cisek P, Neyedli HF, Song JH, Welsh TN (2019). No one knows what attention is. Attention, Perception, & Psychophysics.

[CR25] Kingstone A (2009). Taking a real look at social attention. Current Opinion in Neurobiology.

[CR26] Kuhn G, Kingstone A (2009). Look away! Eyes and arrows engage oculomotor responses automatically. Attention, Perception, and Psychophysics.

[CR27] Lakens D, Caldwell AR (2021). Simulation-based power analysis for factorial analysis of variance designs. Advances in Methods and Practices in Psychological Science.

[CR28] Langton SRH, Bruce V (1999). Reflexive visual orienting in response to the social attention of others. Visual Cognition.

[CR29] Langton SRH, Bruce V (2000). You must see the point: Automatic processing of cues to the direction of social attention. Journal of Experimental Psychology: Human Perception and Performance.

[CR30] McKay KT, Grainger SA, Coundouris SP, Skorich DP, Phillips LH, Henry JD (2021). Visual attentional orienting by eye gaze: A meta-analytic review of the gaze-cueing effect. Psychological Bulletin.

[CR31] Müller HJ, Rabbitt PMA (1989). Reflexive and voluntary orienting of visual attention: Time course of activation and resistance to interruption. Journal of Experimental Psychology: Human Perception and Performance.

[CR32] Pavan G, Dalmaso M, Galfano G, Castelli L (2011). Racial group membership is associated to gaze-mediated orienting in Italy. PLoS One.

[CR33] Peirce J, Gray JR, Simpson S, MacAskill M, Höchenberger R, Sogo H, Kastman E, Lindeløv JK (2019). PsychoPy2: Experiments in behavior made easy. Behavior Research Methods.

[CR34] Posner MI (1980). Orienting of attention. Quarterly Journal of Experimental Psychology.

[CR35] Taylor TL, Klein RM (2000). Visual and motor effects in inhibition of return. Journal of Experimental Psychology: Human Perception and Performance.

[CR36] Troje NF, Westhoff C (2006). The inversion effect in biological motion perception: Evidence for a “life detector”?. Current Biology.

[CR37] Vicovaro M, Dalmaso M, Bertamini M (2022). Towards the boundaries of self-prioritization: Associating the self with asymmetric shapes disrupts the self-prioritization effect. Journal of Experimental Psychology: Human Perception and Performance.

[CR38] Villani C, D’Ascenzo S, Scerrati E, Ricciardelli P, Nicoletti R, Lugli L (2022). Wearing the face mask affects our social attention over space. Frontiers in Psychology.

[CR39] Wang L, Yang X, Shi J, Jiang Y (2014). The feet have it: Local biological motion cues trigger reflexive attentional orienting in the brain. NeuroImage.

[CR40] Zhang X, Dalmaso M, Galfano G, Castelli L (2023). Tuning social modulations of gaze cueing via contextual factors. Psychonomic Bulletin & Review.

